# Foot-and-mouth disease virus non-structural protein antibody detection: from DIVA serology to repeated-epitope design and multi-species field surveillance

**DOI:** 10.3389/fcimb.2026.1894038

**Published:** 2026-07-08

**Authors:** Yang Bai, Kaili Gao, Siqi Wang, Shuchang Cheng, Luyu Mao, Yiming Chang, Lianmin Li, Yongli Guo, Mingchun Gao

**Affiliations:** 1 Heilongjiang Provincial Key Laboratory of Zoonosis, Department of Preventive Veterinary Medicine, College of Veterinary Medicine, Northeast Agricultural University, Harbin, Heilongjiang, China; 2 Department of Immunology, School of Basic Medical Sciences, Harbin Medical University, Harbin, Heilongjiang, China

**Keywords:** 3B/VPg, DIVA, ELISA, foot-and-mouth disease virus, multi-species surveillance, non-structural protein, repeated-epitope design, risk-based surveillance

## Abstract

Surveillance for foot-and-mouth disease (FMD) in vaccinated populations requires tests that distinguish vaccine-induced immunity from infection-associated exposure. Structural protein antibodies help assess vaccine response and serotype-specific protection, whereas antibodies to foot-and-mouth disease virus (FMDV) non-structural proteins (NSPs) support DIVA (differentiating infected from vaccinated animals) interpretation only when vaccine purity and vaccination history are considered. Residual NSPs in incompletely purified vaccines, repeated vaccination, antibody kinetics, host species and assay cut-offs all influence interpretation. This Mini Review examines NSP antibody detection based on 3ABC, 3B/VPg repeated-epitope design, complementary NSP targets, competitive or blocking ELISA, multi-species-compatible assays and emerging field-deployable formats. It does not rank platforms as universally superior; instead, it considers reported performance, validation context and field setting. NSP serology is most useful when combined with vaccination records, structural protein serology, RT-qPCR and epidemiological investigation. It can flag possible subclinical exposure or post-outbreak risk, but it is not a standalone test for current shedding, persistent infection or carrier status.

## 1 Introduction: the DIVA challenge in vaccinated FMD control systems

Foot-and-mouth disease (FMD) is a highly contagious disease of cloven-hoofed animals and remains a major transboundary threat to livestock production, animal movement and trade (Grubman and Baxt, 2004). Laboratory confirmation is essential because field observation alone cannot define infection status or guide all control decisions (Alexandersen et al., 2003). Vaccination-based control programs also depend on surveillance, movement control, outbreak response and maintenance or recovery of FMD-free status (WOAH, 2024). In vaccinated settings, the key serological question is not simply whether antibodies are present, but what type of antigenic exposure they record in relation to vaccination history and herd-level risk (WOAH, 2022).

The diagnostic roles of SP and NSP antibody assays should be separated rather than treated as competing approaches. FMDV structural protein (SP) antibodies follow both vaccination and natural infection, and SP-based assays, including capsid-antigen ELISAs and virus neutralization tests, are used mainly to assess vaccine response, immune coverage and serotype-specific protection. Differentiating infected from vaccinated animals (DIVA) serology asks a different question: whether an antibody pattern provides evidence compatible with viral replication or infection-associated exposure in a vaccinated population (King et al., 2015).

FMDV NSPs provide the main serological contrast for this purpose. During infection, viral replication exposes the host to proteins involved in polyprotein processing, genome replication and virus-host interaction. However, this contrast is only reliable when the vaccine background is considered. Highly purified inactivated vaccines are expected to contain little or no residual NSP, whereas incompletely purified vaccines or repeated vaccination can generate NSP reactivity that complicates DIVA interpretation. The 3ABC polyprotein was established early as a diagnostic antigen for distinguishing infected from vaccinated cattle (De Diego et al., 1997). Recombinant NSP panels broadened the antigenic basis for DIVA testing (Mackay et al., 1998), and baculovirus-expressed 3D, 3AB and 3ABC further supported this approach (Sorensen et al., 1998). Comparative evaluation later confirmed both the utility and the practical limits of NSP ELISAs in vaccinated populations (Brocchi et al., 2006).

The same biology also sets the limits of interpretation. NSP antibody positivity may indicate recent or previous infection exposure, residual NSPs in vaccine preparations, repeated-vaccination background or assay-specific reactivity. Early infection can also be missed before seroconversion. The signal is useful for identifying subclinical exposure or silent circulation in vaccinated herds, but it cannot establish persistent infection, carrier status or active virus shedding on its own. These distinctions require vaccination records, clinical observations, molecular testing, animal movement history and herd-level epidemiology.

This review does not attempt to catalog all FMD diagnostic methods. It focuses on how NSP antibody testing is used in multi-species, field-oriented surveillance by connecting three strands of published work: classical 3ABC DIVA serology, 3B/VPg repeated-epitope design and multi-species-compatible detection formats. The emphasis is practical interpretation for field decision-making, not replacement of official diagnostic protocols.

## 2 Biological rationale and diagnostic boundaries of NSP-based DIVA serology

The rationale for NSP-based DIVA serology is the different antibody profile expected after vaccination and after viral replication. FMDV SPs, including VP1, VP2, VP3 and VP4, form the capsid and contain major neutralizing antigenic sites. Inactivated FMD vaccines are designed to present these structural antigens. SP antibodies therefore provide useful information on vaccine-induced immunity and serotype-specific protection, but they cannot by themselves separate vaccination from infection.

NSPs are different because they are produced during viral replication. FMDV proteins such as L, 2A, 2B, 2C, 3A, 3B/VPg, 3C and 3D participate in polyprotein processing, genome replication, membrane remodeling and host-cell modulation. In natural infection, the host may mount antibody responses to both structural and non-structural antigens. In animals given highly purified inactivated vaccines, NSP exposure should be absent or very limited. This contrast explains why NSP antibodies can mark infection-associated immune exposure in vaccinated populations.

Because NSPs are less tied to capsid antigenic variation, NSP assays can support broadly serotype-independent screening for infection-associated antibody responses. That advantage has a clear boundary. NSP antibody detection does not identify whether exposure involved serotype O, A, Asia 1, SAT 1, SAT 2, SAT 3, C or particular lineages. Virus typing, sequencing, vaccine matching and neutralization assays remain necessary when serotype or antigenic relationship matters for outbreak response or vaccine selection (WOAH, 2022).

In practice, each diagnostic layer answers a different question. SP antibody assays and virus neutralization tests mainly inform immunity and serotype-specific protection. RT-qPCR detects viral genome and is most useful during early or active infection. NSP antibody assays add evidence of infection-associated immune exposure, particularly where SP antibody results are difficult to interpret because vaccination is widespread. These tools are complementary, not interchangeable.

An NSP-positive result should not be treated as direct evidence of current infectiousness. It records an immune response compatible with viral replication or NSP-containing antigenic background, but it does not by itself distinguish recent infection, past infection, persistent infection or current virus shedding. Because NSP antibody persistence varies with species, infection history, assay format and sampling time, a single positive result should not be used to infer when infection occurred without longitudinal or virological evidence. The FMDV carrier state remains a virological and epidemiological problem rather than a serology-only diagnosis (Stenfeldt and Arzt, 2020). Conversely, an animal sampled soon after infection may be RT-qPCR positive but NSP antibody negative because seroconversion has not yet occurred.

The distinction matters most in low-prevalence or post-control settings. Even highly specific assays can produce results that need confirmation when the expected prevalence is low. A single weak positive in a vaccinated herd has a different meaning from clustered NSP reactivity among epidemiologically linked animals. Validation should also consider possible cross-reactivity or non-specific reactivity associated with other vesicular disease agents or related picornavirus exposures when such exposure is epidemiologically plausible; this issue is less consistently documented than vaccine-related NSP background. Positive, equivocal or borderline results should be read together with vaccine batch information, SP antibody levels, RT-qPCR results, clinical observations, movement records and herd-level context.

## 3 NSP targets and assay platforms for infection-associated antibody detection

Work on FMDV NSP antibody detection has followed two practical questions: which antigen best captures infection-associated exposure, and which assay format remains reliable under surveillance conditions? Early DIVA studies centered on recombinant NSPs, especially 3ABC. Later work moved in two related directions: repeated-epitope or combined-antigen design to improve infection-associated recognition, and multi-species-compatible assay formats to support surveillance across host species. ELISA platforms remain the technical base for both directions (Ma et al., 2011). **Table 1** summarizes the main NSP targets, assay formats, reported performance values and validation boundaries. The DSn and DSp values quoted in the table come from separate validation panels, antigen preparations and cut-off definitions, and should be read as study-specific estimates rather than direct platform rankings.

**Table 1:** FMDV NSP targets and assay formats for DIVA surveillance.

Target or platform	Representative validation evidence	Reported diagnostic performance	Practical interpretation	Main limitations/field readiness
3ABC ELISA/recombinant 3ABC	Established DIVA antigen; representative studies include De Diego et al. (1997); Brocchi et al. (2006); Lu et al. (2007), and Zia et al. (2022).	Example: r3ABC in-house ELISA, DSn 95.3% and DSp 96.3% in a characterized validation panel (Zia et al., 2022). Confirmatory NSP ELISAs detected about 90% of exposed vaccinated carrier cattle with ~99% specificity (Tewari et al., 2021).	Routine laboratory DIVA screening and vaccinated-herd exposure assessment.	Performance depends on vaccine purity, vaccination history, infection stage, cut-off, host species and expected prevalence. Positive results require confirmation in low-prevalence settings.
3B/VPg repeated-epitope ELISA	Repeated 3B epitope peptide ELISA evaluated in cattle (Gao et al., 2012).	At cut-off 0.3: sensitivity 96.8%; specificity 99.8% in naive cattle and 99.0% in vaccinated cattle serum panels (Gao et al., 2012).	Repeated-epitope antigen design for DIVA assay development; complementary to 3ABC.	Not a true multi-epitope platform unless different epitopes are combined. Validation is mainly cattle-focused and should not be generalized without further species testing.
3D/2C complementary targets	Evaluated as part of recombinant NSP panels or baculovirus-expressed antigen sets (Mackay et al., 1998; Sorensen et al., 1998).	Data lacking for routine standalone field use. Standardized DSn/DSp ranges based on field sample panels are not established; available values remain study- and panel-dependent.	Candidate complementary antigens for multi-target or confirmatory assay design; not routine standalone screening.	Research-stage or complementary use. Insufficient evidence for routine field surveillance; requires comparative and multi-laboratory validation.
Conventional indirect ELISA	Widely used laboratory format; 3ABC indirect ELISAs include Lu et al. (2007); Hosamani et al. (2015), and Zia et al. (2022).	Representative r3ABC indirect ELISA: DSn 95.3% and DSp 96.3% in a characterized validation panel (Zia et al., 2022). Protein G-based multi-species-compatible indirect ELISA was evaluated using domestic host serum panels (Hosamani et al., 2015).	Large-scale laboratory testing in defined species when conjugates and cut-offs are validated.	Species-specific secondary antibodies or variable Ig-binding can limit broad use. Cut-offs and background reactivity require host-specific validation.
Competitive/blocking ELISA	3ABC monoclonal blocking ELISA (Sorensen et al., 2005); nanobody 3ABC competitive ELISA (Gelkop et al., 2018); 10H9 3B blocking ELISA (Hosamani et al., 2022).	Nanobody competitive ELISA: DSn 94% and DSp 97.67% in a cattle serum validation panel (Gelkop et al., 2018). 10H9 bELISA: DSn 95%, DSp 98%, cut-off PI >50% in known positive/negative sera (Hosamani et al., 2022).	Potentially useful for multi-species-compatible screening when the competing reagent, cut-off and host-specific validation are available.	Performance depends on monoclonal/nanobody specificity, antigen configuration, cut-off and validation panel. Multi-species use remains conditional on host-level validation and is not automatically superior to indirect ELISA.
Ig-binding protein-based ELISA	Protein G or engineered Ig-binding detection reduces dependence on species-specific conjugates (Hosamani et al., 2015; Chang et al., 2026).	Published studies support multi-species-compatible feasibility, but performance values are assay- and species-dependent and are not directly interchangeable across hosts or assay designs.	Candidate host-range-adapted serology for multi-species-compatible FMD monitoring when host-specific cut-offs and validation panels are available.	Ig-binding differs by species and IgG subclass. Requires host-specific cut-offs, field panels and independent reproducibility testing; equivalent performance across hosts should not be assumed.
Emerging rapid or field-deployable formats	Lateral-flow or fluorescence immunoassay formats have potential for front-line triage. One recent LFA comparison with indirect ELISA used 32 stored cattle samples (Jauhari et al., 2024).	Limited peer-reviewed NSP-specific field data. Example LFA versus indirect ELISA: sensitivity 95.2% and specificity 100% in 32 stored cattle samples (Jauhari et al., 2024), but robust cross-species field DSn/DSp ranges with sample-size and CI reporting are lacking.	Triage tool for border, farm, market or post-outbreak screening only when linked to confirmatory laboratory testing and epidemiological follow-up.	Should not be treated as a standalone diagnostic basis. Performance is comparator-, operator-, sample-, species- and setting-dependent; positive or equivocal field results require laboratory serology, RT-qPCR and epidemiological interpretation.

DSn, diagnostic sensitivity; DSp, diagnostic specificity; PI, percent inhibition; CI, confidence interval. Reported DSn and DSp values are quoted from the original validation studies and should not be interpreted as direct head-to-head comparisons across platforms. For in-house assays, estimates depend on antigen preparation, coating or expression conditions, conjugate or competing reagent, cut-off criteria, validation-panel composition and host species. Sample sizes, panel information or CIs are shown where reported; values not reported in the original publications were not inferred. The term multi-species-compatible indicates a design or validation aim and does not imply equivalent diagnostic performance across susceptible host species.

Among NSP targets, 3ABC remains the reference point for DIVA serology. Its use for distinguishing infected from vaccinated cattle was established in early ELISA studies (De Diego et al., 1997). Recombinant NSP panels later broadened the antigenic basis of DIVA testing (Mackay et al., 1998), and baculovirus-expressed 3D, 3AB and 3ABC antigens further supported the principle (Sorensen et al., 1998). Comparative and validation studies confirmed the value of 3ABC-based ELISAs while showing that performance depends on assay format and epidemiological setting (Brocchi et al., 2006; Lu et al., 2007). Recent work continues to place emphasis on validation rather than antigen choice alone (Zia et al., 2022).

3B/VPg offers a narrower, epitope-focused route. As a replication-associated NSP, it contains conserved linear B-cell epitopes that can be arranged as repeated-epitope antigens. A repeated 3B epitope peptide-based ELISA distinguished FMDV-infected cattle from vaccinated cattle and serves as an example of repeated-epitope NSP antigen design (Gao et al., 2012). This design should be described as repeated-epitope design rather than a true multi-epitope platform unless different epitopes are combined. Repeated-epitope designs are best viewed as complements to 3ABC, not replacements for it, because 3ABC has a broader validation history.

Other NSPs, including 3D and 2C, have also been tested as diagnostic antigens. The 3D polymerase is relatively conserved, whereas 2C and other NSPs may add antibody signals that complement 3ABC or 3B-based assays. The field is unlikely to converge on a single universally superior antigen. A more useful goal is to combine antigens in ways that balance sensitivity, specificity, conservation, manufacturability and interpretability.

Conventional indirect ELISA remains attractive because it is mature, scalable, inexpensive relative to many platforms and compatible with routine laboratory workflows. It is effective for large-scale testing in a defined domestic species. Its weakness is the need for species-specific secondary antibodies. That requirement becomes inconvenient when surveillance must include cattle, sheep, goats, pigs, buffalo or wildlife-related samples (Hosamani et al., 2015).

Competitive and blocking ELISAs address part of this limitation by measuring the ability of serum antibodies to compete with or block defined monoclonal antibody binding. A 3ABC monoclonal antibody-based blocking ELISA illustrates this approach for DIVA testing (Sorensen et al., 2005). Nanobody-based competitive ELISA and multi-species-compatible blocking ELISA show how defined-reagent systems can extend NSP detection across species (Gelkop et al., 2018; Hosamani et al., 2022). Their performance still rests on antibody quality, epitope selection, cut-off setting and host-species validation.

Recombinant antigens have moved NSP assays away from crude viral preparations and toward safer, more standardized production. Epitope-engineered antigens are a further refinement. By arranging conserved infection-associated epitopes as repeated or combined constructs, they aim to improve antigen consistency and targeted recognition. Conservation alone, however, is not enough. Antibody accessibility, antigen conformation, host species, expression system, purification quality, background reactivity, cost and cold-chain requirements all influence diagnostic performance and implementation.

Multi-species-compatible assay design is a practical requirement for FMD surveillance. Field programs may involve cattle, sheep, goats, pigs, buffalo and other susceptible hosts. Ig-binding protein-based ELISA reduces dependence on species-specific conjugates and represents a shift from antigen-centered design toward host-range-adapted detection (Chang et al., 2026). Together with 3B/VPg repeated-epitope ELISA, this work illustrates how antigen design and detection format can be developed in parallel. The term multi-species-compatible still requires caution because Ig binding differs among species and IgG subclasses, and each host species needs its own validation context. It should be read as a design or validation aim, not proof of equivalent performance in all susceptible species.

Emerging rapid or field-deployable NSP antibody formats, including lateral-flow and fluorescence immunoassays, may support screening at farms, borders, markets and post-outbreak monitoring sites. A recent comparison of an anti-NSP lateral-flow assay with indirect ELISA reported 95.2% sensitivity and 100% specificity in 32 stored cattle samples from Indonesia (Jauhari et al., 2024), but this result is assay-, comparator- and panel-specific rather than broad field validation. These formats should therefore be regarded as triage tools rather than final diagnostic methods. Published validation for NSP-specific rapid tests remains more limited than for laboratory ELISA, and performance can be affected by sample quality, operator technique and threshold interpretation. Positive or equivocal field results should lead to confirmatory laboratory serology, RT-qPCR when appropriate and epidemiological investigation.

In brief, indirect ELISAs are scalable and familiar but often depend on species-specific or Ig-binding conjugates. Competitive or blocking ELISAs reduce some host-species constraints but rely on a defined competing reagent and cut-off. Repeated-epitope designs improve antigen standardization but require comparative validation. Rapid formats improve access, but their role should remain triage until broader field validation is available.

## 4 From DIVA testing to multi-species and field-oriented surveillance

**Figure 1:**
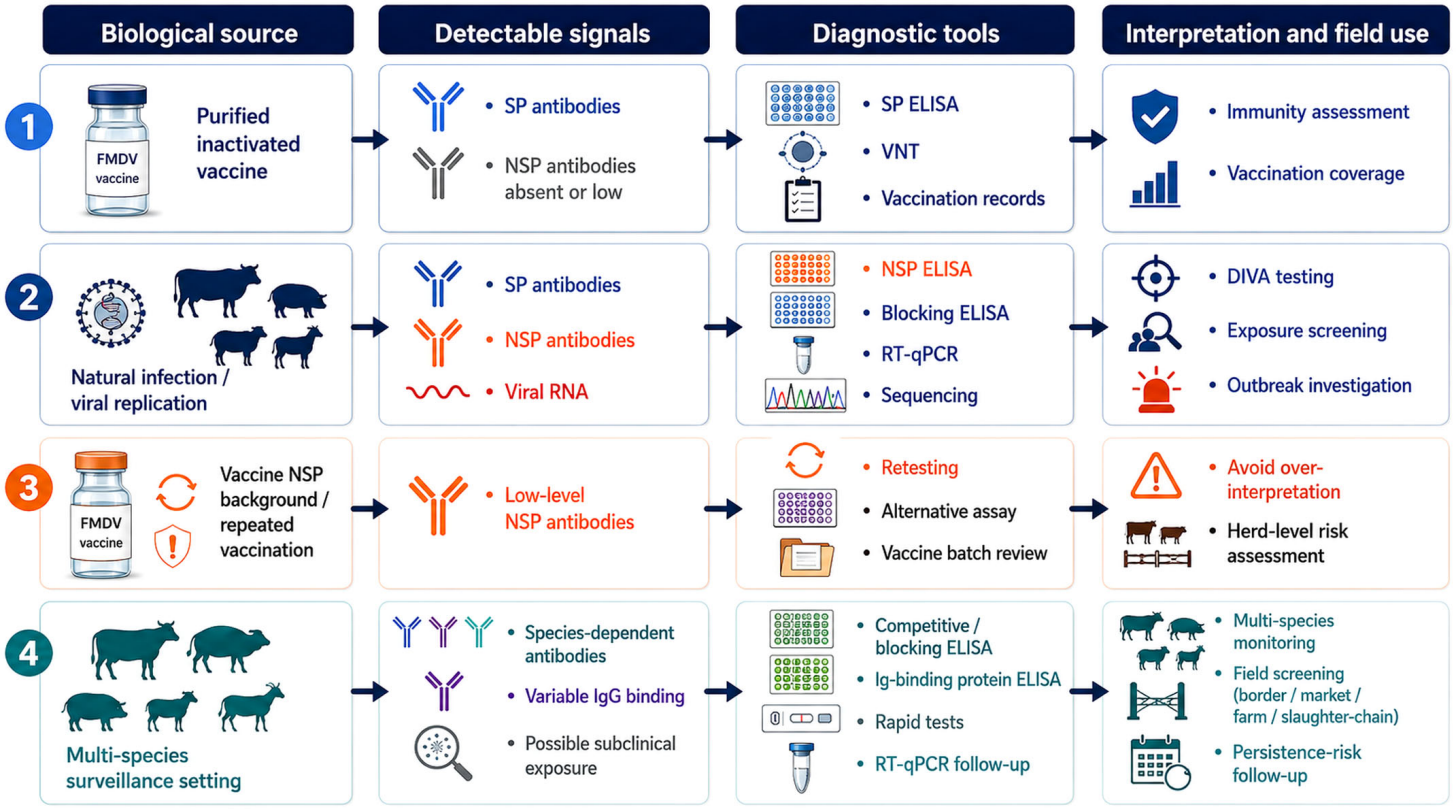
Scenario-based interpretation framework for FMDV NSP antibody detection in vaccinated and multi-species surveillance settings. The figure summarizes four practical scenarios: purified vaccination, natural infection or viral replication, vaccine-related NSP background or repeated vaccination, and multi-species surveillance. It links biological source, detectable signals, diagnostic tools and field-use interpretation. The framework is a scenario-based guide, not a formal decision tree or official diagnostic algorithm. NSP results should be interpreted with vaccine purity and vaccination history, assay format, host species, RT-qPCR or other molecular confirmation, and herd-level epidemiological evidence. DIVA, differentiating infected from vaccinated animals; NSP, non-structural protein; RT-qPCR, reverse transcription quantitative PCR; VNT, virus neutralization test.

The value of FMDV NSP antibody detection depends on how the result is used. In the laboratory, NSP serology separates infection-associated antibody responses from vaccination-induced SP responses. In surveillance, the result is an exposure signal that must be interpreted with vaccination history, RT-qPCR, clinical information and herd-level epidemiology. This distinction is important in vaccinated regions, where control depends on detecting exposure without treating every serological reaction as proof of active circulation. **Figure 1** summarizes a scenario-based interpretation framework across purified vaccination, natural infection or viral replication, vaccine-related NSP background and multi-species surveillance settings.

Because FMDV vaccine protection is largely serotype- and strain-dependent, NSP serology should be interpreted as exposure-oriented rather than serotype-identifying. It can support broadly serotype-independent screening for infection-associated antibody responses, but virus typing, sequencing, vaccine matching and neutralization tests remain necessary when serotype or antigenic relationship matters for outbreak response or vaccine selection (WOAH, 2022, WOAH, 2024).

FMD is also a multi-species disease. Cattle, swine, sheep, goats, buffalo and other cloven-hoofed hosts differ in susceptibility, clinical expression and epidemiological relevance. Mixed farms, livestock markets, border areas, communal grazing systems, wildlife-livestock interfaces and slaughter-chain monitoring may all require more than one target species. A diagnostic format optimized for a single host can be limiting in these settings.

Assay format therefore becomes a surveillance issue. Conventional indirect ELISA may work well in one defined species but still require species-specific conjugates. Competitive, blocking and Ig-binding protein-based formats reduce that dependence and can simplify multi-species-compatible testing. Even so, compatibility across host species cannot be assumed from reagent design alone. Species differ in immunoglobulin structure, IgG subclass distribution, antibody kinetics, sample background and non-specific reactivity; each relevant host needs validation with characterized vaccination, infection and field panels. Direct head-to-head comparisons between multi-species-compatible NSP ELISAs and species-optimized assays using the same characterized serum panels remain limited. Multi-species-compatible claims should therefore be presented as validation requirements, not as assumed platform properties.

NSP results are most useful at the herd or population level. A single positive result in a vaccinated animal may reflect prior exposure, vaccine-related NSP background, non-specific reactivity or a true infection-associated response. Without clinical, molecular or epidemiological support, it should prompt confirmation rather than immediate control conclusions. Clustered NSP reactivity across pens, herds or linked groups carries greater epidemiological weight and justifies expanded investigation (Tewari et al., 2021).

NSP serology can also reveal exposure that is clinically unapparent. In vaccinated herds, infection may be mild, subclinical or partly masked by immunity. NSP antibodies can flag herds in which viral replication may have occurred despite the absence of obvious disease. This is useful for post-outbreak follow-up and for screening possible silent circulation. It does not prove persistent infection or carrier status. Persistence requires virological investigation, appropriate upper aerodigestive tract sampling when relevant, repeated molecular testing and epidemiological tracing (Stenfeldt and Arzt, 2020). In practice, NSP serology should trigger targeted follow-up; it is not a standalone carrier test.

In field use, recurring result patterns require different levels of follow-up without imposing a universal decision threshold. An NSP-negative, RT-qPCR-negative and SP-positive vaccinated herd is consistent with vaccine-induced immunity and no detected infection-associated exposure, within the limits of sampling. An isolated NSP-positive but RT-qPCR-negative animal calls for retesting, alternative-assay confirmation and review of vaccine history. Clustered NSP positivity points to a stronger exposure signal and should lead to expanded sampling and molecular testing. NSP-negative but RT-qPCR-positive results may reflect early infection before seroconversion, while combined NSP and RT-qPCR positivity requires immediate epidemiological investigation according to national control policy.

Risk-based FMD surveillance works by combining these signals. SP antibody assays and virus neutralization tests inform vaccine-induced immunity and serotype-specific protection. NSP assays add evidence of infection-associated exposure, including possible subclinical exposure. RT-qPCR detects viral genome, especially in early or active infection. Virus isolation, sequencing and antigenic characterization support confirmation, serotype identification and vaccine matching. Clinical signs, vaccination records, movement history and herd contacts supply the context. In this workflow, FMDV NSP antibody detection functions as one layer of field-oriented surveillance rather than as a stand-alone laboratory endpoint.

## 5 Discussion: from DIVA serology to risk-based surveillance

FMDV NSP antibody detection is most useful when embedded in risk-based surveillance, not when a stronger serological signal is treated as an endpoint. NSP serology remains a retrospective exposure marker, subject to window periods, low-titre or equivocal reactions, and false-positive risks in low-prevalence or repeatedly vaccinated populations. The first priority is standardized validation and interpretation of existing NSP assays. This includes defined serum panels, transparent cut-offs, vaccine-purity information, repeated-vaccination backgrounds and confirmatory pathways for low-prevalence settings. Stronger signal alone is not enough; sensitivity, specificity, positive predictive value and operational consequences must be considered together.

The second priority is validation of multi-species-compatible assays. Classical 3ABC assays will remain important, but complementary targets such as 3B/VPg, 3D and 2C may broaden detection across infection stages, host species or antibody-response patterns. Repeated-epitope 3B/VPg designs can improve antigen standardization, but they need to be evaluated against 3ABC-based assays using comparable panels. Multi-species-compatible assays should be tested across cattle, pigs, sheep, goats, buffalo and other relevant hosts with host-specific cut-offs and field samples.

The third priority is field-deployable formats and connected surveillance. Rapid tests can support front-line screening during outbreaks, at borders, in markets, on farms and in slaughter-chain surveillance, but they should not be presented as mature substitutes for laboratory ELISA until field validation is stronger. Useful field deployment requires confirmation procedures, linkage to vaccination and movement records, integration with RT-qPCR and clear rules for retesting or escalation.

NSP serology fits best inside risk-based surveillance systems. These systems should combine vaccine batch and immunization records, SP antibody levels, NSP antibody results, RT-qPCR, clinical observations, movement data, herd history and regional outbreak context. The purpose is graded follow-up, from routine monitoring to retesting, expanded sampling and, where required by national policy, movement control or outbreak investigation.

In conclusion, FMDV NSP antibody detection is no longer only a laboratory DIVA tool. In vaccinated and multi-species settings, its value comes from integration with RT-qPCR, vaccination records and herd-level epidemiological investigation, rather than from isolated serological interpretation. Further progress will depend on standardized validation, host-specific interpretation, confirmatory pathways and cautious deployment of field-oriented formats.
